# Extraction of pH-Dependent DNA-Binding Anti-Tumoral Peptides from *Saccharomyces cerevisiae*

**DOI:** 10.3390/ph19010184

**Published:** 2026-01-21

**Authors:** Francesco Ragonese, Loretta Mancinelli

**Affiliations:** 1 Department of Chemistry, Biology and Biotechnologies, University of Perugia, Via Elce di Sotto 8, 06123 Perugia, Italy; loretta.mancinelli@libero.it; 2 Department of Human Sciences and Quality of Life Promotion, San Raffaele University of Rome, Via di Val Cannuta, 247, 00166 Rome, Italy

**Keywords:** small peptide, peptide extraction, anti-tumoral peptide, cancer, drug discovery, cell cycle, DNA damage

## Abstract

Cancer remains a significant challenge in the field of medicine, primarily due to its inherent plasticity and the development of resistance to conventional therapeutic interventions. Genomic mutations and the activation of oncogenes enable cancer cells to resist senescence and apoptosis, leading to uncontrolled growth with harmful consequences. Small peptides are molecules with interesting anti-tumour properties and represent a valid alternative to conventional treatments. Our group has previously identified a class of small peptides bound to the DNA that can be extracted from the chromatin of various tissues, including wheat germ and trout. These peptide pools have been shown to possess interesting antiproliferative and apoptotic properties, and they are associated with cell cycle regulation. However, given the complexity of the extraction process, it is necessary to identify a substrate that will enable a more efficient extraction of these peptides, while also ensuring a composition that is simple to investigate. The present study developed a method for the extraction of this group of peptides from yeast, and the extract was then tested on cancer cells in order to confirm its anti-tumoral properties. The peptides were obtained from chromatin extracted from *Saccharomyces cerevisiae* cells through alkalisation and purification by gel filtration chromatography. The extract was tested on HeLa cells to verify its effects on vitality and the cell cycle. The data demonstrate that the chromatographic profile of this peptide extract indicates a more basic composition than the pool extracted from other tissues and exhibits comparable antiproliferative properties. The ability to rapidly obtain a biologically active, analytically accessible, and adequately purified fraction from the widely available substrate *Saccharomyces cerevisiae* represents a significant advance in the study of these DNA-binding peptides.

## 1. Introduction

It is well known that cancerogenesis is a multistep process governed by mutations at the level of different genes, which results in the gain of function of oncogenes and a loss of function of tumour suppressors. Epigenetic mechanisms, which are heritable, can be responsible for the dysfunction of gene expression regulation. This, in turn, can lead to the inactivation of tumour suppressor genes [1,2]. The loss of genomic integrity results in the inability of the cell to control proliferation and induce programmed cell death or senescence [3]. The result of this process is abnormal cell proliferation, which is the hallmark of cancer. The system has been described as heterogeneous, containing distinct cell populations that possess the capacity to replace cells that have been destroyed by chemotherapy. This replacement is facilitated by the presence of redundant subpopulations within the system [4]. This “cancer robustness” theory suggests that therapy strategies should be directed to cellular dynamics rather than focusing on a single target. For instance, the genetic instability that is characteristic of cancer cells has been utilized to impede the proliferation of tumour cells. In fact, research indicates that cancer cells are more sensitive to genetic damage than normal cells, due to genetic mutations at the level of genes involved in DNA repair and proliferative activity. Ionizing radiations and most of the chemotherapeutic drugs, acting by DNA damage induction, show a higher effectiveness toward cancer cells with respect to normal cells [5]. In many cases, however, the heterogeneity of the cancer cell population allows certain cells to survive by engaging redundant pathways that regulate cell proliferation and migration. In this view, the use of mixture compounds instead of single molecules could represent a useful therapy strategy to overcome relapses observed in clinical responses [6]. In addition, it has been observed that various hallmark traits of cancer cells are regulated by the same oncogenes. These oncogenes are responsible for generating a wide range of different types of cancer cells. This small set of genes and related pathways could represent potential targets for developing new anticancer agents [7].

Many natural product-derived mixtures have been reported to target the machinery controlling cell proliferation [8]. Among them, small peptide (SP) extracts from natural sources such as plants or animal tissue have attracted considerable interest due to their interesting anticancer properties, such as high absorption, minimal toxicity, and ease of modification and synthesis. These bioactive peptides are typically composed of 10–50 amino acids. The mechanisms through which SPs exert their effects against tumour cells include the induction of apoptosis, the disruption of membranes, the inhibition of angiogenesis, the modulation of signalling pathways, and immunomodulation [9,10,11]. Their small size, specificity, and flexibility allow them to target cancer cells while minimizing damage to healthy tissue. In comparison to antibodies, they exhibit superior tissue penetration and stability. Their ability to cross biological barriers and low immunogenicity further enhance their therapeutic potential [12].

In previous studies, we described a class of small DNA-binding peptides with a molecular weight of approximately 1000 Da. These peptides have been shown to inhibit tumour cell proliferation in vitro by inducing DNA damage, cell cycle arrest, and apoptosis [13,14]. These peptides represent the lower-molecular-weight component obtained by alkaline treatment from deproteinised DNA, which still contains protein material despite treatment with NaCl, chloroform, isoamyl alcohol, and phenol. The presence of these peptides is likely attributable to their attachment to DNA via bonds that are not ionic or hydrophobic, which are typically impacted by conventional purification methods [15]. They have been found in the chromatin of various eukaryotic tissues (calf thymus, bull spermatozoa, trout testis, wheat germ pea, and wheat sprout) and characterized by a pH-dependent binding to DNA that decreases from pH 7.5 to 9 [16,17]. The quantity obtained from the DNA of cancer cells is significantly lower than that obtained from the corresponding normal cells [18]. The biological effects shared by this family of chromatin peptides and their presence in various tissues indicate that they could be involved in a conserved mechanism of cell growth. Their lower presence in cancer cells as compared to normal cells also suggests that such a putative control mechanism may be lost in neoplastic transformation; so far, they could represent a new potential factor in the approach to cancer treatment. To improve the knowledge about their effects at the molecular level, it is fundamental to identify the sequences belonging to the peptide pool. The main difficulties are represented by the complexity of the extraction procedure and the small amount of purified peptide pool available to be subjected to structural investigation (*w*/*w* ratio peptides/DNA ranges from 0.001 to 0.03). Previous studies have attempted to characterize these extracts more precisely, but synthetic peptides constructed on the basis of mass spectroscopy data were only effective on in vitro transcription systems and not on cells [19]. Spectrometry analysis performed on a peptide mixture rather than on single sequences is complicated and does not allow the obtainment of enough information regarding the exact sequences. In order to overcome these issues, it is necessary to consider alternative extraction sources that allow the development of a simpler extraction procedure and the obtainment of a less complex peptide mixture. Yeast, and in particular *Saccharomyces cerevisiae*, is known to be a source of small peptides with various bioactive properties. These can act as antioxidants, antimicrobials, and possibly even anticancer agents [20]. Given the simplicity of the structures and biological processes of yeast cells, we hypothesised that a simpler set of peptides could be obtained from *Saccharomyces cerevisiae* chromatin than from the DNA of more complex eukaryotic cells. In addition, it was assumed that the peptide pool could be purified more rapidly.

In the present study, the presence of this class of DNA-binding peptides in yeast cells was investigated. A dedicated extraction method has been developed, and the biological effect of the yeast chromatinic peptide extract (YCPE) was examined to ascertain its similarity to those found in the DNA of wheat germ, which have been utilized as extraction sources to date.

## 2. Materials and Methods

### 2.1. Reagents and Materials

Except where otherwise specified, all reagents come from Sigma-Aldrich (Merck, Darmstadt, Germany). For cell cultures, all necessary medium and supplements were purchased from Euroclone (Pero, Italy).

### 2.2. Peptide Extraction from Wheat Germ and Yeast Chromatin

The extraction and purification of peptides from wheat germ were performed as described in [15]. For the extraction of peptides from *Saccharomyces cerevisiae,* commercial yeast (Lievital, Lesaffre Italia, Trecasali, Italy) was used. After freezing and thawing, 100 g of product was boiled for 40 min under constant stirring in 144 mL of distilled H_2_O with 5.5 g of Sodium dodecyl sulphate (SDS) and then centrifuged at 9500× *g* for 20 min at 4 °C. The supernatant was retrieved and mixed under agitation with 5% SDS (*v*/*v* in water) and 1 M NaCl for a period of 20 min at room temperature. Thereafter, an equivalent volume of chloroform was added and stirred overnight. Then, the mixture was centrifuged at 9500× *g* for 15 min at 4 °C and the supernatant was mixed with two volumes of cold ethanol and left at −20 °C overnight. After further centrifugation, the pellet obtained was washed three times in 70% ethanol, resuspended in absolute ethanol, and left at −20 °C overnight. The pellet was recovered by centrifugation at 7800× *g* at 4 °C for 20 min. This method enabled the extraction of approximately 100 mg of nucleic acids from the initial 100 g of product. The pellet was then resuspended in water and mixed with an equal volume of 0.4 M ammonium acetate at pH 9.5 adjusted with ammonia 30% for 30 min at room temperature. This step ensures the separation of the peptide fraction from the DNA. The extracted peptides were collected by adding two volumes of cold ethanol, leaving the mixture overnight and then centrifuging it at 7800× *g* at 4 °C for 20 min to remove any residues. The mixture was then lyophilized.

### 2.3. Gel Filtration Chromatography

The lyophilized peptide obtained through extraction was resuspended and purified by size-exclusion gel filtration chromatography using Sephadex G25 as the stationary phase and acetic acid 1% as the mobile phase, Column calibration was performed using dextran blue for void volume determination and bacitracin, oxytocin, oxidized glutathione, and potassium dichromate as molecular weight references (Supplementary Figure S1). The peptide fraction was collected at the peak corresponding to a molecular weight of 1000 Da with an elution volume equal to Ve/V0 = 2. The value of the ratio was obtained from the results of four different extractions. After column purification, the YCPE was lyophilized before testing it on cells.

### 2.4. Thin Layer Chromatography and Peptide Quantification

The YCPE was subjected to acidic hydrolysis with HCl 37% *v*/*v* for 3 h at 110 °C under vacuum conditions. Silica plates for TLC (Merck, Darmstadt, Germany) were loaded with 2.5 and 1 μg/mL of hydrolysed extract and subsequently eluted with a solution comprising n-butanol, pyridine, acetic acid, and distilled water, with a volumetric ratio of 6:2:3:3 for 2.5 h. Next, the dried plates were stained with a solution of 50 mM of ninhydrin (2,2-diidrossi-1,3-diossoidrindene) in acetic acid and ethanol (1:10 *v*/*v*) to identify the peptide portions. To determine the concentration of the extracted peptide pool, a densitometry analysis was carried out using ImageJ software (ver1.54h). The 2 mM solutions of serine, threonine, and phenylalanine were used as a reference for ninhydrin coloration and densitometry quantification.

### 2.5. Cell Culture

HeLa cells were obtained from ATCC (American Type Culture Collection, Manassas, VA, USA) and cultivated in 25 cm^2^ flasks (Falcon, Corning, Glendale, AZ, USA) with phenol-red Dulbecco’s Modified Eagle Medium (DMEM) with 100 IU/mL penicillin/streptomycin, 10% fetal bovine serum, and 200 mM L-glutamine. The cells were incubated at 37 °C in 5% CO_2_ humidified atmosphere, and the medium was changed twice a week. Cells were subcultured when they reached 80% of confluence with Trypsin/EDTA 0.025%.

### 2.6. Vitality Assay

To assess the effect of the extract on the viability of HeLa cells, the MTT assay was performed. In order to ensure the optimal growth of cells, 5 × 10^3^ cells/well were plated with complete medium in a 96-well plate (Falcon, Corning, Glendale, AZ, USA). After 24 h from seeding, the cells were exposed to different concentrations of YCPE (1, 2, 4, 8, 16 µg/mL), resuspended in sterilized water, and added directly to the culture medium. Cells without treatment were used as a control. After 24 h, cells were incubated with 0.5 mg/mL of 3-(4,5-dimethylthiazol-2-yl)-2,5-diphenyltetrazolium bromide (MTT) for 3 h; then, the medium was removed, and 200 µL of Dimethyl Sulfoxide (DMSO) was added to the wells. The analysis was conducted at a wavelength of 550 nm using a Varian Cary 100 scan spectrophotometer (Agilent, Santa Clara, CA, USA). The results of six technical replicates of three independent experiments were expressed as a normalized percentage based on the comparison of the absorbances of the treated cells with those of the untreated control cells.

### 2.7. Cell Cycle

Cells were plated at a density of 1 × 10^5^ cells/cm^2^ onto a 25 cm^2^ flask and subsequently exposed to 1 or 6 µg/mL of YCPE for a period of 24 h. After incubation, the cells were trypsinized, washed with ice-cold phosphate-buffered saline (PBS), and fixed in 80% ethanol overnight and then placed at −20 °C until cell cycle analysis was conducted. Nuclei were isolated from fixed cell suspensions and stained with propidium iodide (50 μg/mL) for the determination of DNA content. The samples were analyzed using a FACS Calibur laser flow cytometer (Becton Dickinson, San Jose, CA, USA). In total, 10,000 events were acquired for each sample. Percentages of cells in the G_1_, S, and G_2_M phases of the cell cycle were quantified using WinCycle software 1.2 (Phoenix Flow Systems, San Diego, CA, USA).

### 2.8. Statistical Analysis

All statistical analyses were performed using Prism Graph Pad 9 software. Data are expressed as the mean ± standard deviation (SD) and analyzed using a one-way ANOVA test in combination with Dunnett’s multiple comparison test. *p* < 0.05 (*), *p* < 0.01 (**), *p* < 0.001 (***), and *p* < 0.0001 (****) were used to assess the significance of the results.

## 3. Results

### 3.1. Extraction of Peptides

**Figure 1:**
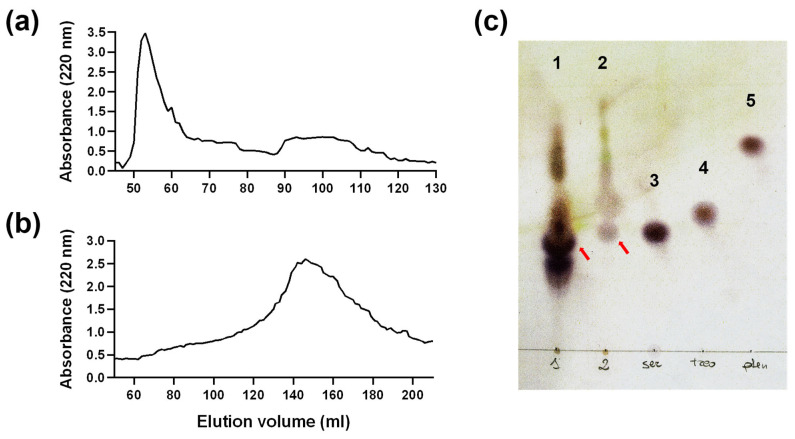
Chromatography profile of YCPE (**a**) and wheat germ extract (**b**) on Sephadex G25; the fraction corresponding to Ve/V0 = 2 corresponds to 120–180 mL for YCPE and 90–120 for wheat germ extract. (**c**) TLC chromatography of the YCPE. Spots 1 and 2 indicate YCPE spots (2.5 and 1 μg/mL), and 3 to 5 indicate the reference standard amino acids (3-serine, 4-threonine, 5-phenylalanine) used as a positive control for ninhydrin staining. Red arrows indicate the spots used for calculate the Rf for sample 1 and 2. Rf for the samples was 0.74 and 0.77 for YCPE 2.5 (1) and 1 μg/mL (2), 0.75 for serine (3), 0.71 for threonine (4), and 0.57 for phenylalanine (5).

The chromatographic profile of the yeast fraction obtained by alkalinisation in conjunction with spectrophotometric reading at 220 nm of the extracted nucleic acids shows the presence of a peak at Ve/V0 = 2.04 ± 0.25, which corresponds to the molecular weight of 1000 Da according to column calibration (Figure 1b and Supplementary Figure S1). This weight corresponds to that of the peptides extracted from wheat germ chromatin (Figure 1b). These also elute at twice the empty volume when extracted under identical elution conditions. The protein nature of the extract was evaluated by staining with ninhydrin after TLC chromatography. The ninhydrin reaction to the primary and secondary amino groups of amino acids and peptides results in the formation of coloured spots, which can be visualised on thin-layer chromatography (TLC) plates (Figure 1c). A shift in the lower spots is evident in sample 2 compared to sample 1. This phenomenon could be attributed to the presence of an artefact, given that the concentration of sample 1 is higher than that of sample 2. This discrepancy in concentrations may have hindered the effective migration process. It can be theorised that the lowest spot in sample 2 encompasses the two lowest spots in sample 1. Therefore, the calculation of the Retention Factor (Rf) was performed by taking into consideration the centre of the spots indicated by the red arrows. The concentration of the peptide pool was evaluated by comparing the densitometric values of the YCPE samples with those obtained for known amounts of reference amino acid loaded on the same plate. The concentration of the extracted peptides was found to be approximately 100 μg/mL, while the total material recovered from 100 g of yeast was approximately 200 μg.

### 3.2. Effect of the Extract on Cell Viability

**Figure 2:**
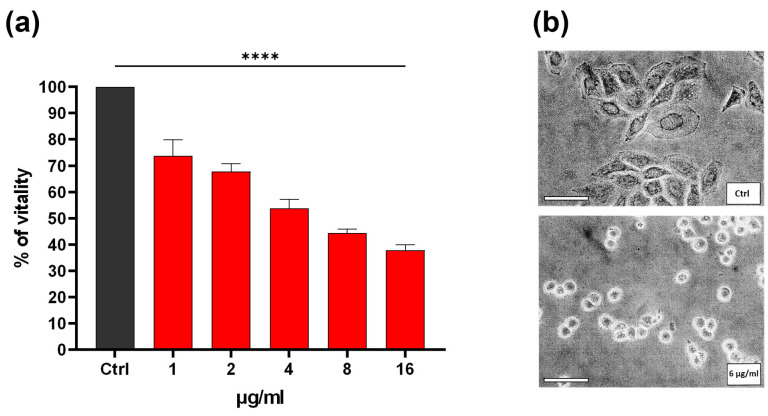
(**a**) Bar plot of MTT viability assay of HeLa cells treated with different concentrations of YCPE. The bars represent the mean values of at least three experiments plus SD. **** *p* < 0.0001 indicates the significance obtained with Dunnett’s multiple comparison test of treated cells (red) vs. control (dark gray). (**b**) Representative images of control cells (upper) and cells treated with 6 μg/mL of YCPE (lower). Scale bar: 100 μm.

The effect on tumour cell viability of the YCPE fraction recovered at Ve/V0 = 2 was investigated. For this purpose, HeLa cells (human cervical cancer cells) were employed. The cells were exposed for 24 h to various concentrations of extract, ranging from 1 to 16 μg/mL and subjected to the MTT test. As demonstrated in Figure 2a, a substantial and dose-dependent decline in viability was observed at all concentrations evaluated, with an IC50 of approximately 6 μg/mL. This decline in viability is accompanied by a significant morphological change in the cells, shifting from a polygonal to a rounded configuration (Figure 2a).

### 3.3. Evaluation of the Effects of the YCPE on the Cell Cycle

**Figure 3:**
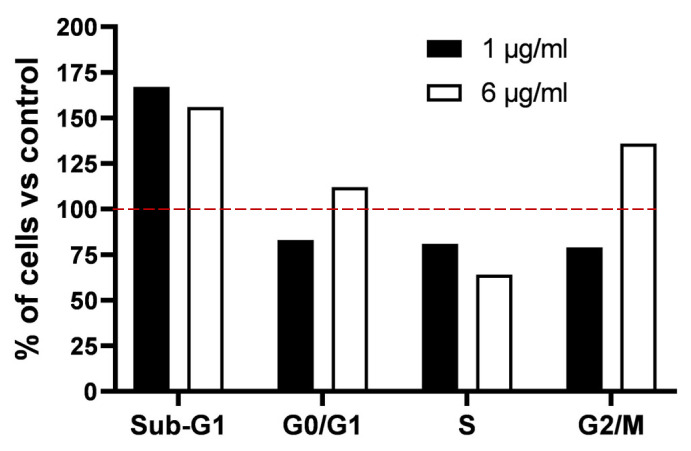
Bar graph representing the percentage of cells in the various phases of the cell cycle following 24 h of treatment with 1 and 6 μg/mL of YCPE. Each bar represents the percentage change in the number of treated cells compared to the number of cells in the corresponding phase of the cell cycle of the untreated control group. The red dashed line indicates the value 100% as a reference point to facilitate the visualization of the results.

In order to assess alterations in the cell cycle, HeLa cells were exposed to a concentration of 1 and 6 μg/mL of the extract for a period of 24 h. The administration of 1 μg/mL of the YCPE resulted in a significant increase in the number of cells in the sub-G1 phase of about 65%, whilst concomitantly reducing the number of cells in the other phases by approximately 17, 19, and 21% for G0/G1, S, and G2/M, respectively (Figure 3, black bars). In the case of the 6 μg/mL treatment, cytofluorimeter analysis revealed an accumulation of cells in the G2/M phase of approximately 36%, accompanied by a 37% decrease in cells in the S-phase compared to the control. The cells in the G0/G1 phase exhibited a slight positive variation, approximately 12%. The data therefore appear to indicate G2-phase cell cycle arrest and a substantial increase in the sub-G1 phase of approximately 56%, which is consistent with the previously measured decrease in vitality (Figure 3, white bars).

## 4. Discussion

Carcinogenesis is a multistep process driven by genetic mutations that activate oncogenes and inactivate tumor suppressor genes, as well as by heritable epigenetic alterations disrupting gene expression. These changes impair genomic integrity, leading to uncontrolled cell proliferation and resistance to apoptosis or senescence. Tumor heterogeneity and the presence of redundant cellular subpopulations contribute to cancer’s robustness, enabling survival and relapse following conventional therapies [21]. Consequently, targeting cellular dynamics, rather than single molecular entities, has been proposed as a more effective therapeutic strategy [6]. Among the numerous natural product-derived treatments, SPs from plant or animal sources have gained attention for their anticancer properties. Due to their small size, specificity, and low toxicity, SPs offer advantages over traditional therapies, including better tissue penetration, reduced immunogenicity, and enhanced therapeutic efficacy [10].

In our previous studies, we demonstrated that chromatin preparations derived from different types of eukaryotic cells, after extensive deproteinization, still contain peptidic material that can be detached by further treatment with an alkaline buffer [16,17]. Subsequent fractionation of this material resulted in the identification of a pool of low-molecular-weight peptides of approximately 1000 Da that, independently of the source of extraction, exert interesting biological effects. Their removal by alkaline treatment from the DNA of normal cells enhances the DNA template capacity, while such an effect is lower or absent in several cancer cell lines [22]. Their extraction level from the DNA of cancer cells is significantly lower than that found in the DNA of the corresponding normal cells [18]. This indicates that such a class of peptides could be involved in a mechanism that may be lost during carcinogenesis, while their ubiquitous presence suggests that such a hypothetical mechanism is highly conserved. The peptide pool, extracted from wheat germ chromatin, induces growth inhibition in Hela cells when the cells are treated during the S phase only and is associated with DNA damage, activation of the G2 checkpoint control pathway, G2 arrest of the cell cycle, and caspase-dependent apoptosis [13,14]. It is therefore possible to hypothesize that this class of peptides can restore the ability, lost by tumor cells, to undergo the apoptotic process, and that it could represent a potential therapeutic factor for the treatment of cancer. Nevertheless, the structural configuration of effector molecules remains unclear. The primary challenges are the complex extraction of these peptides and obtaining properly isolated sequences for mass spectrometric analysis. This is due to the fact that the peptide pool consists of sequences with highly correlated amino acid composition [19].

The aim of this study was to identify an alternative source that would facilitate the development of a more efficient extraction procedure using *Saccharomyces cerevisiae* cells, known to be a source of interesting bioactive peptides [20]. This approach was motivated by the assumption that, given the high level of conservation of these peptides in different types of tissues, they might also be present in simpler eukaryotic cells, such as yeast cells. We also considered that this type of cell might offer the advantage of a faster extraction procedure and a mixture of peptides with less complex sequences, which would be easier to separate and identify. To obtain results comparable to those of the reference studies, we used the same methods for the extraction and characterization of the peptides.

The findings presented in this study demonstrate that it is possible to extract a pool of peptides of approximately 1000 Da from a matrix by alkali treatment of the chromatin fraction. Indeed, the obtained fraction was found to possess a peptidic nature and a molecular weight that was similar to that of the fractions obtained from wheat germ chromatin. Furthermore, a comparison of the Sephadex chromatographic profiles obtained under identical elution conditions of YCPE and the peptide pool extracted from wheat germ chromatin reveals that the profile of the yeast extract excludes the presence of higher-molecular-weight molecules. This makes the process of extracting peptides from yeast chromosomes faster than the process used for wheat germ. This is due to the fact that it allows further fractionation steps to be circumvented. In addition, the reduced number of bands in the thin-layer chromatographic profile suggests a lower degree of variability in peptide sequences. This could facilitate the subsequent identification of individual components. Nevertheless, the yield of this extraction process remains relatively low (~200 µg per 100 g yeast). This low concentration of the peptide pool may result from their probable function as regulators rather than structural components. Furthermore, the DNA was extensively deproteinized prior to alkaline treatment, which may have contributed to the low yield, that can vary from different extractions.

The anti-tumoral effects of YCPE on HeLa cells are similar to those previously observed for peptides extracted from wheat germ. YCPE has been demonstrated to exert a dose-dependent effect on the viability of cells and promote G2/M-phase blockade presumably as a result of DNA damage, as also suggested by previous studies [13]. These effects, in conjunction with a significant increase in Sub-G1 phase cells, suggest an induction of apoptosis resulting from a block in the cell cycle. Based on the experience of previous work, it is likely that signal proteins such as Chk1 (checkpoint kinase 1) play a role in the mechanisms that result in cycle blockade and apoptosis. This is a marker of G2 checkpoint activation, which prevents cells with genomic damage from entering mitosis and is often activated following DNA damage [23]. The block of the cycle leads to an apoptotic process that could occur through a caspase-dependent mechanism due to the involvement of caspases 7 and 9, an increase in the pro-apoptotic protein Bax (Bcl-2-associated X protein), and a decrease in the anti-apoptotic protein Bcl-2 (B-cell lymphoma 2). This may indicate an unsuccessful attempt to repair DNA damage [24]. However, the possibility of alternative mechanisms, such as the interaction of peptides with cell membranes, which may yield analogous outcomes in terms of apoptosis and toxicity, cannot be excluded [25]. Furthermore, the presence of contaminants such as wall residues from yeast cells, which may contribute to the toxicity mechanism of the extract, cannot be excluded.

This study demonstrates that it is possible to obtain a pool of peptides after alkalinization of yeast’s DNA with chromatographic and anti-tumour properties similar to those that can be obtained from wheat germ. However, the degree to which peptide structures are similar between different species remains to be verified. To ascertain the sequences and molecular mechanisms triggered by YCPE, an in-depth analysis is required. This will necessitate a further separation of the peptide pool, the identification of the sequences that exert the greatest biological effect, and the study of their anti-tumoral properties on tumour cells using more advanced methods and analysis. Moreover, it will be necessary to verify and overcome issues typical of nuclear targeting peptides, such as poor bio-distribution, premature degradation in biological fluids, and transport methods to the nucleus [26,27]. In view of this, the capacity to obtain a sufficiently purified, biologically active, and easily analysable fraction from a widely available substrate, such as *Saccharomyces cerevisiae*, promises a more profound understanding of the anti-tumour potential of these chromatin peptides.

## Data Availability

The original contributions presented in this study are included in the article/Supplementary Materials. Further inquiries can be directed to the corresponding author.

## Supplementary Materials

The following supporting information can be downloaded at: https://www.mdpi.com/article/10.3390/ph19010184/s1, Figure S1. Chromatography column calibration. Column calibration plot on Sephadex G25. Dextran Blue was used for Void Volume (V0) determination. Standard used for calibration was Bacitracin (1422 Da; Ve/V0 = 1.32), Oxitocin (1007 Da; Ve/V0 = 2.02), Oxidized glutathione (GSSG; 612 Da; Ve/V0 = 2.37) and potassium dichromate (294 Da; Ve/V0 = 2.65).
