# Supplementary material for: Extraction of pH-Dependent DNA-Binding Anti-Tumoral Peptides from Saccharomyces cerevisiae
Source: Pharmaceuticals (Basel). 2026 Jan 21;19(1):184. doi: 10.3390/ph19010184 (PMC12845435; doi:10.3390/ph19010184)
Supplement: Supplementary file 1 [file pharmaceuticals-19-00184-s001.zip › pharmaceuticals-4055324-supplementary.pdf]

Extraction of pH-dependent DNA-binding anti-tumoral peptides from *Saccharomyces cerevisiae.*

Supplementary files

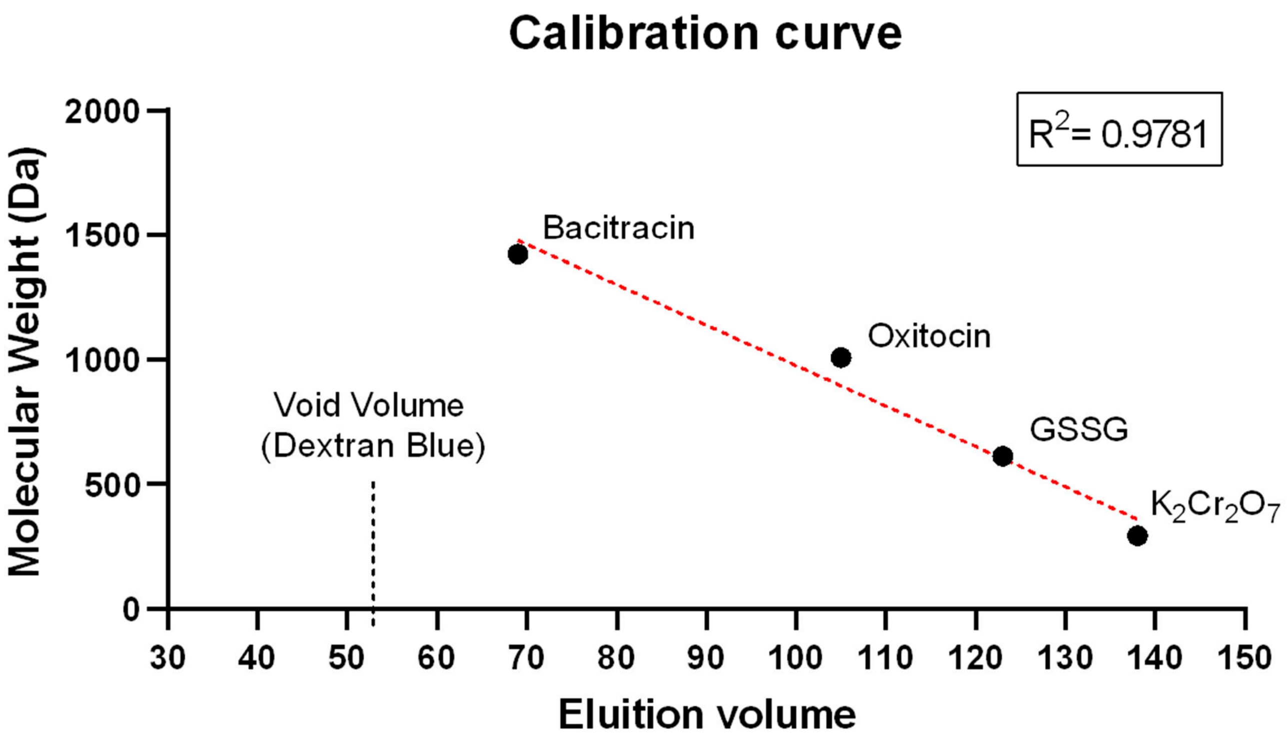

Supplementary Figure S1. Chromatography column calibration. Column calibration plot on Sephadex G25. Dextran Blue was used for Void Volume (V0) determination. Standard used for calibration was Bacitracin (1422 Da; Ve/V0=1.32), Oxitocin (1007 Da; Ve/V0=2.02), Oxidized glutathione (GSSG; 612 Da; Ve/V0=2.37) and potassium dichromate (294 Da; Ve/V0=2.65).
